# The Most Common Affected Body Regions in Breakdancers: A Descriptive Epidemiological Study in Italy

**DOI:** 10.3390/jfmk11010073

**Published:** 2026-02-12

**Authors:** Pierpaolo Panebianco, Aurora Trovato, Marco Sapienza, Francesca Locatelli, Francesco Leonforte, Rosario Ferlito, Vito Pavone, Gianluca Testa

**Affiliations:** 1 Department of General Surgery and Medical-Surgical Specialties, A.O.U. Policlinico Rodolico-San Marco, University of Catania, 95123 Catania, Italy; pierpaolo.panebianco@gmail.com (P.P.); auroratrovato98@gmail.com (A.T.); marcosapienza09@yahoo.it (M.S.); francesca.locatelli1254@gmail.com (F.L.); gianpavel@hotmail.com (G.T.); 2 Department of Integrated Hygiene, Organizational, and Service Activities (Structural Department), Health Management, University Hospital Policlinic “G.Rodolico-San Marco”, 95123 Catania, Italy; francesco.leonforte@policlinico.unict.it; 3 Department of Biomedical and Biotechnological Sciences, University of Catania, 95123 Catania, Italy; ferlito.rosario@libero.it

**Keywords:** breakdance injuries, hip hop injuries, breakdancing, musculoskeletal injuries, breakers, overuse

## Abstract

**Background:** This study aims to characterize the musculoskeletal injury landscape among Italian adolescent and adult breakdancers, specifically evaluating the correlation between technical execution and various risk factors. We conducted a cross-sectional analysis on a cohort of 97 practitioners (68 professionals and 29 amateurs). Data were retrieved using the “Breakdance Injury Questionnaire” (BIQ), a specialized 28-item tool covering training volume, clinical history, and technical specialization. **Results:** The data reveal a striking injury burden, with an overall prevalence rate of 94.84%. The most frequent sites of injury were the knee (63.9%), shoulder (60.8%), and wrist (57.7%). A significant statistical disparity in injury risk was observed between professionals and amateurs (*p* = 0.037), with amateurs exhibiting a higher vulnerability to acute trauma. Of clinical note is the significant correlation between intensive powermoves practice and shoulder pathology (*p* = 0.029). Conversely, generic preventive measures, including standard warm-ups (*p* = 0.168) and protective equipment (*p* = 0.164), showed no significant efficacy in reducing trauma incidence. **Conclusions:** Breakdancing is a high-demand discipline with a traumatic profile comparable to elite gymnastics. The functional inversion of the upper limbs predisposes athletes to specific overuse syndromes. Future prevention strategies must focus on specific conditioning protocols and qualified coaching rather than generic warm-up routines.

## 1. Introduction

Breaking, globally recognized as “breakdancing,” is a dynamic urban dance form that originated in the socio-economically disadvantaged neighborhoods of New York City, specifically the Bronx, during the early 1970s. Over the decades, what began as a niche street subculture has evolved into a highly athletic competitive discipline requiring a unique combination of artistic expression and extreme physical performance, until achieving full Olympic recognition with his official debut at the 2024 Paris Olympics [1].

A comprehensive understanding of the injury mechanisms in breaking requires a detailed analysis of the technical progression of the dance, which is traditionally composed of four essential elements performed in a continuous sequence [1,2,3].

Breaking, globally recognized as a cultural art form with significant athletic elements, is characterized by a multifactorial performance model that integrates physical, artistic, and strategic resources [4]. As a primary subset of the broader “street dance” category—defined as an urban dance form rooted in public spaces for self-expression and community interaction—breaking is classified as a classic “Old School” hip-hop style that emerged between the 1970s and 1980s [5]. The routine typically commences with “Toprocks,” movements performed in an upright position that serve as the introductory phase. These steps, heavily influenced by African American dance styles and adapted from Latin dances such as Salsa and Rumba, primarily require rhythmic coordination and agility in an open kinetic chain [6]. Within the competitive “battle” format, dancers must adapt these movements to unknown music provided by a DJ and respond to the opponent’s performance in a “question and answer” dynamic [4,5]. As the routine intensifies, the dancer transitions to the floor to execute “Footworks” (or floorworks). In this phase, the athlete supports their body weight primarily through the upper extremities while performing intricate circular movements with the legs and pelvis [2,7]. Biomechanically, this shifts the upper limbs into a closed kinetic chain function—a weight-bearing role for which the human shoulder and wrist are not evolutionarily adapted—requiring significant muscular strength and joint stability [8].

The complexity of the performance escalates with powermoves which represent the most acrobatic and technically demanding component of breaking. These dynamic maneuvers involve continuous rotation of the body derived from momentum, requiring explosive power, balance, and extreme range of motion [9]. Physiologically, these moves contribute to a high cardiorespiratory demand; elite breakers exhibit a VO^2^ peak significantly higher than that of dancers in traditional genres like ballet or contemporary dance [10]. During these sequences, the dancer often pivots on the head, shoulders, or elbows, subjecting these anatomical structures to high repetitive axial loads and rotational shear forces [11]. Consequently, persistent practice of these overhead movements is strongly associated with the development of overuse injuries and specific tendinopathies [6]. The sequence is frequently punctuated or concluded by “Freezes,” static poses used to accentuate the musical beat. In a freeze, the dancer halts movement abruptly, often suspending the body in unstable, inverted positions for several seconds, necessitating maximal isometric contraction and superior motor control to maintain balance against gravity [9,12]. This discipline is classified as a high-intensity intermittent exercise, where short rounds of approximately 2 min generate significant levels of blood lactate [10]. To execute this repertoire, which includes jumps, rapid directional changes, and acrobatic tricks, the breaker must possess physiological attributes comparable to those of an elite gymnast, including explosive power, agility, and high anaerobic capacity. However, unlike gymnasts, many breakdancers lack formal coaching and structured conditioning programs. This discrepancy between the high biomechanical demand and the often-autodidactic training method makes this population highly susceptible to both acute traumatic injuries and chronic musculoskeletal disorders [1,13]. Despite the global proliferation of breaking, epidemiological research remains sparse [14].

Currently, there are limited studies investigating the injury patterns in this specific cohort, and no epidemiological data exist regarding the Italian population. Previous international studies have highlighted the high prevalence of musculoskeletal injuries; specifically, Cho et al. analyzed 144 Korean professional breakdancers, reporting that 95.2% had sustained at least one injury, with the wrist (69.4%), fingers (61.1%), and knee (55.6%) being the most affected areas [12]. Similarly, Kauther et al. examined 40 practitioners (mean age 21.1 years), finding that 95% of professionals and 85% of amateurs sustained injuries, identifying the knee (24%) and wrist (19%) as primary injury sites [13]. These data, alongside the French study by Cousin and Poisson, provide a robust baseline for comparison [15]. Furthermore, comparisons with general hip-hop dance studies, such as those by Ojofeitimi et al., suggest that breakers face a significantly higher injury incidence due to the specific acrobatic nature of the discipline [1,16]. Therefore, the objective of this descriptive epidemiological study is to investigate the most affected anatomical regions among Italian breakdancers. By analyzing a sample of professional and amateur dancers, this research aims to identify the prevalence of injuries and correlate them with endogenous and exogenous risk factors, thereby filling a significant gap in the current orthopedic literature.

## 2. Materials and Methods

### 2.1. Study Design

This descriptive epidemiological investigation was designed as a cross-sectional study. The research protocol aimed to assess the prevalence, anatomical distribution, and risk factors associated with musculoskeletal injuries in the Italian breakdancing community. The data collection period spanned from April 2024 to May 2025. Data collection was conducted using a structured survey administered through multiple channels: digital platforms (social media and email) and on-site recruitment as well as direct data collection during the “Check the Style” international breaking competition held in L’Aquila, Italy.

### 2.2. Participants and Selection Criteria 

A non-probability convenience sampling strategy was employed for this study, resulting in an initial target population that consisted of 117 dancers (44 amateurs and 73 professionals) recruited from various regions across Italy. While a formal power analysis for sample size calculation was not performed prior to recruitment, the study aimed to enroll as many eligible participants as possible to establish a robust baseline for this specific athletic niche. To ensure the homogeneity of the sample, subjects were included if they met specific requirements: Italian nationality, active practice for at least 6 months, a minimum training volume of 3 h per week, and age between 12 and 46 years. 

A critical distinction was made between “professional” and “amateur” status, a classification often complex in the breaking community due to its autodidactic roots. In this study, “professional” status was defined by the dancer’s primary source of income, participation in international-level competitions, or affiliation with professional dance companies. Conversely, “amateurs” were defined as those practicing the discipline for recreation or local competition without a primary financial or contractual commitment to the sport. This distinction is essential, as professionals often demonstrate superior neuromuscular control but are exposed to higher cumulative chronic loads [13].

To minimize confounding variables related to other athletic activities with distinct injury profiles, candidates practicing classical dance concurrently were excluded from the study. Following the screening process based on these criteria, a total of 20 participants were excluded, resulting in a final sample size of 97 valid responses eligible for statistical analysis.

### 2.3. Data Collection

Data collection was conducted using an anonymous online questionnaire specifically designed for this research via the Google Forms platform. 

Participants were recruited through a multi-channel strategy aimed at capturing a heterogeneous population: the “Breakdance Injury Questionnaire” (BIQ), an anonymous 28-item online tool structured into three logical sections (demographics, training habits, and clinical history). The first section collected general demographic and anthropometric data, such as sex, age, weight, and height, to characterize the study population. The second section investigated training habits and extrinsic factors, including the type of flooring used, supervision by a coach, and the frequency of protective equipment usage, which was assessed using a 5-point Likert scale ranging from “never” to “always”. The final section focused on the dancers’ clinical history, gathering detailed information on past or present injuries sustained during their career. For precise anatomical localization, injuries were categorized into 11 specific districts—head, neck, spine, shoulders, elbows, wrist, hand, hip, knee, ankle, and foot—following the methodological approach validated in previous reference literature [13,14,17].

For the purposes of this study, an “injury” was defined as any musculoskeletal complaint—either acute traumatic or chronic overuse—sustained during training or competition that resulted in the need for medical consultation, a modification of training methods, or a suspension of athletic activity. Injuries were classified into 11 specific anatomical districts: head, neck, spine, shoulders, elbows, wrist, hand, hip, knee, ankle, and foot. This classification follows the methodological approach validated in previous orthopedic and dance-specific literature, allowing for standardized comparison with international cohorts [13].

The inclusion criteria ensure that participants have acquired fundamental technical skills (foundations) and are subject to relevant biomechanical loads, consistent with established urban dance research models. Conversely, exclusion criteria were established to minimize confounding variables: subjects with incomplete data and those concurrently practicing classical ballet or other highly codified dance forms were excluded [10]. This methodological choice is supported by the need to distinguish breaking’s injury profile—characterized by acrobatic loads and closed kinetic chain functions of the upper limbs—from styles with radically different biomechanical demands, such as ballet [4].

The online questionnaire was shared across major social media groups dedicated to the Italian breaking scene and via direct email campaigns to regional dance hubs. Physical recruitment and digital flyers were distributed in specialized urban dance centers and sports clubs across the national territory to capture both high-level athletes and local practitioners. Direct data collection was performed by the research team (A.T. and F.L.) during the “Check the Style” international breaking competition in Aquila, Italy, ensuring the inclusion of elite-level practitioners. The dissemination process was supervised by the senior authors (G.T. and V.P.), while the primary investigation and participant outreach were managed by the investigators (A.T. and F.L.). 

Finally, the sample of 97 valid dancers was categorized into “professional” and “amateur” groups.

### 2.4. Statistical Analysis

The collected data were exported and organized using Microsoft Excel for initial descriptive analysis. Continuous variables were expressed as means and standard deviations, while categorical variables were reported as frequencies and percentages. To ensure the validity of our parametric comparisons, the normality of continuous data was assessed using the Shapiro–Wilk test prior to inferential analysis. Inferential statistical analysis was performed to identify significant associations between risk factors and injury occurrence, employing the Student’s t-test for continuous variables and the Chi-square test of independence for categorical variables, such as the comparison between professional and amateur status or the correlation between specific moves and injury sites. A *p*-value of less than 0.05 was considered statistically significant for all analyses. 

## 3. Results

### 3.1. Demographic Characteristics and Training Habits 

The final study cohort comprised 97 Italian dancers, predominantly male (82 subjects, 84.5%) compared to females (15 subjects, 15.5%). The mean age of the participants was 26.09 ± 8.08 years, with a mean Body Mass Index (BMI) of 22.60 kg/m^2^, reflecting a physically active population with normal body mass index. Regarding training volume, the sample consisted of highly active individuals: 72.16% of respondents reported training for more than five hours per week, while the remaining 27.84% trained between three and five hours. Statistical analysis revealed no significant correlation between training volume (hours/week) and injury incidence (*p*-value = 0.68), suggesting that exposure time alone is not the sole determinant of trauma in this cohort.

There are no significant differences in age, height, and weight between professional (68 Dancers) and amateur dancers (29 Dancers) (Table 1). Inclusion criteria required participants to have a minimum of six months of breakdancing experience and a training frequency of no less than three hours per week. Specifically, 27 dancers (14 professionals) train between 3 and 5 h weekly, while the remaining 70 participants (54 professionals) train for a minimum of 5 h per week. The data obtained were analyzed, and the results show no statistically significant correlation between those who train 3 h per week and those who train 5 h regarding a higher likelihood of injury (*p* = 0.68). Participants were also asked whether they engage in other activities alongside breaking: 48 individuals, 35 of whom are professionals, do not practice any other discipline, while 49 participants (18 professionals) do. The results show no significant correlation between those who only practice dance and the presence of injuries (*p* = 0.5107).

### 3.2. Injury Prevalence and Anatomical Distribution

The clinical history of our cohort underscores a substantial injury burden, with 94.84% of the participants reporting at least one dance-related trauma throughout their career. Among the eleven anatomical districts analyzed, the knee emerged as the most vulnerable joint, followed closely by the shoulder and the wrist. While the knee was consistently the most affected site regardless of proficiency level, our analysis revealed that professional status appears to exert a relative protective effect; professionals demonstrated a significantly lower overall injury risk compared to their amateur counterparts (*p* = 0.037). Granular incidence rates and the complete anatomical distribution for both groups are detailed in Table 2.

### 3.3. Protection, Warm-Up, and Training Supervision

Analysis of injury prevention habits revealed that 96.9% of the cohort (*n* = 94) consistently performed a warm-up before training, while only three participants reported omitting this step. Similarly, post-session stretching was a common practice, utilized by 87.6% of the dancers (*n* = 85). Despite the high prevalence of these routines, statistical analysis showed no significant difference in injury rates between those who warmed up and those who did not (*p* = 0.168). Regarding the use of protective equipment, the data showed significant variability: 34.02% of breakdancers never utilized protection, and 39.17% reported using it only rarely. Conversely, 16.49% used protective gear frequently, and a small minority (10.31%) reported consistent usage during every session.

Additionally, 68 individuals (70.10%), including 48 professionals (49.8%) and 20 amateurs (20.62%), report not being supervised by a coach, while only 29 (29.90%), including 20 professionals (20.62%) and 9 amateurs (9.28%), report training under the supervision of a coach. There is no statistically significant difference between those training with a coach and those who do not (*p* = 0.13).

**Figure 1:**
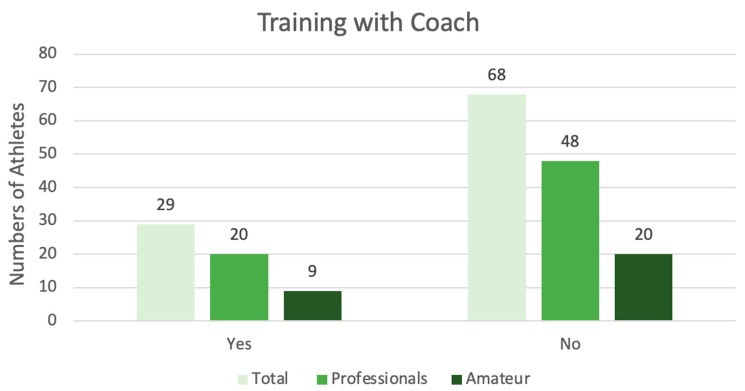
Training with Coach: This table shows the numerical difference between athletes who report training with a coach and those who train independently.

The data collected are summarized in Figure 1 below:

### 3.4. Skills 

The questionnaire revealed that the main skills (toprocks, footworks, powermoves, and freezes) are almost always trained simultaneously. Most participants report doing a complete workout that incorporates all the skills to build as comprehensive a dance entry as possible. Specifically, 73 breakdancers focus more on footworks, 60 on powermoves, 47 on freezes, and 38 on toprocks (Table 3).

The data obtained thus far were analyzed to verify possible statistically significant correlations. The following findings emerged:

There is no significant difference between the skills most trained by professionals and those performed by amateurs (*p* = 0.87).

Those who train powermoves more frequently are more likely to sustain shoulder injuries: these data were statistically significant (*p* = 0.029).

### 3.5. Sex Differences 

The data collected through the questionnaire suggest that, out of a sample of 15 subjects, 3 women train between 3 and 5 h per week, while 12 train more than 5 h weekly. Additionally, 7 of them train independently, while 8 are supervised by a coach. Compared to men, women appear more inclined to have supervision during training (*p* = 0.031).

However, 14 out of 15 women have sustained at least one injury in some body region during their career. The three most affected areas in women due to breakdancing were the knee (66.67%), shoulder (33.33%), and wrist (33.33%) (Table 4). All the female participants perform stretching and warm-up routines. As with the b-boys, for the b-girls, a longer warm-up period does not show a reduced predisposition to injuries (*p* = 0.45).

### 3.6. Circumstances of Injury

Of the total participants in the questionnaire, 7 of them claimed to have been injured during a Contest (competition), 3 during an event, 71 claimed to have suffered trauma during training and the remaining part (11 dancers) “not in a specific context”. 

### 3.7. Training Surfaces

Most of the participants train, depending on the circumstance, on different surfaces. Out of a total of 97 subjects, 72 train on parquet, 13 on concrete, 18 on marble, 30 train on linoleum, 13 on asphalt and 8 on tatami. A very small percentage, such as not to be considered in the overall count, also carries out their workouts on: tiles, laminate, masonite or on tiled surfaces; Finally, about 10 individuals carry out their workouts on all the surfaces mentioned above.

### 3.8. Medical Monitoring and Post-Injury Behavior

While 78.35% of the practitioners sought formal medical consultation after an injury, a smaller segment of 13.40% bypassed professional care, relying instead on self-directed recovery measures without clinical oversight. Among those who sought medical assistance, 46 individuals resumed training at the same level as before the injury, 28 experienced improvements after rehabilitation or medical consultation but had to modify their training methods, and 4 individuals reported unsuccessful rehabilitation.

Out of the 97 participants, 53 stated that they had suspended their training after the injury, whereas 38 continued training despite the pain. The duration of training suspension showed wide variability, ranging from one month to over a year. Notably, approximately 37% of respondents were unable to specify the exact duration of their interruption, leaving this section of the questionnaire incomplete. This substantial proportion of missing data should be considered when interpreting the results, as it limits the completeness of the conclusions regarding post-injury recovery and follow-up. Excluding these individuals, the remaining responses were as follows: 41 dancers reported a training interruption of 1–3 months, 9 reported 3–6 months, 3 reported 6–12 months, and 3 reported more than 12 months.

## 4. Discussion

The present study represents the first epidemiological investigation specifically targeting the breakdancing community in Italy, aiming to fill a significant gap in the national orthopedic literature. The primary finding of this research is the alarmingly high prevalence of musculoskeletal injuries within this cohort. Our data indicate that 94.84% of the analyzed dancers have sustained at least one injury related to their practice during their career. This incidence rate characterizes breakdancing as a discipline with a high traumatic potential, placing it at the upper spectrum of risk among artistic-athletic activities. A direct technical comparison with elite gymnastics is currently constrained by the absence of a standardized severity metric, such as ‘days of absence’ from sport. While both disciplines appear to share similar anatomical injury patterns and biomechanical stressors, the lack of consistent ‘time-loss’ data in our cohort prevents a rigorous quantitative assessment of clinical severity or recovery timelines between these two populations. These findings are strongly consistent with international literature; specifically, our data align closely with the study by Cho et al., which reported an injury rate of 95.2% in a similar Korean cohort [12]. Furthermore, when compared to broader studies on urban dance styles, such as the investigation by Ojofeitimi et al. which reported a 68% injury rate among a mixed population of hip-hop dancers, our results suggest that the specific biomechanical demands of breaking—characterized by acrobatics and floorwork—predispose practitioners to a significantly higher risk profile than other urban dance genres [16].

Complementary to these epidemiological figures, a recent systematic overview of case reports conducted by Brborović et al. provides crucial insight into the clinical severity of these injuries [14]. Synthesizing 50 years of literature, their review highlights that while surveys capture prevalence, case reports reveal a spectrum of severe acute trauma—including fractures and dislocations—and chronic conditions such as nerve compressions, predominantly affecting the upper extremities, head, and neck. Brborović et al. identify high-impact movements during spins and balancing as primary injury mechanisms, further emphasizing the value of detailed medical diagnoses over self-reported data to understand the true pathological burden of the discipline [14].

Regarding the anatomical distribution of injuries, our results identified the knee, shoulder, and wrist as the most frequently affected regions, following a hierarchical pattern that reflects the unique pathophysiology of this discipline. The knee emerged as the most injured joint overall (63.9%), a finding that corroborates the observations of Kauther et al. and Cho et al. [12,13,18]. Biomechanically, this prevalence can be attributed to the repetitive high-impact loading and extreme ranges of flexion required during “Footworks” and landing phases. These movements generate substantial shear forces on the menisci and patellofemoral compressive loads, often executed without the shock absorption provided by footwear appropriate for the training surface [11,15,16,19].

However, the most clinically relevant finding of this study emerges from the analysis of the upper extremities. Unlike traditional dance forms or upright sports, breakdancing inverts the physiological function of the human body, transforming the upper limbs into primary weight-bearing structures. Our statistical analysis revealed a significant correlation (*p*-value = 0.029) between the practice of powermoves and the incidence of shoulder injuries6. This association is supported by a clear biomechanical rationale: the glenohumeral joint, evolutionarily designed for mobility rather than load-bearing, lacks the inherent bony stability of the acetabulum [13]. During powermoves—such as windmills or airflares—the shoulder is subjected to repetitive high-velocity axial compression and rotational torque. These forces place immense stress on the dynamic stabilizers, particularly the rotator cuff and the glenoid labrum, predisposing the athlete to impingement syndromes, tendinopathies, and instability [14,16]. The findings here essentially bridge the gap between anecdotal clinical observation and empirical data. The recorded *p*-value of 0.029 substantiates the correlation between technical powermoves and shoulder injuries, effectively moving the relationship from clinical suspicion to statistically grounded evidence. This correlation strongly supports the earlier classification by Cousin and Poisson, who identified these acrobatic sequences as the most hazardous component of breaking [15]. Biomechanically, this makes sense; we are witnessing the impact of high-velocity torque and axial loading on a joint architecture that was never designed for primary weight-bearing. By quantifying this risk, our study provides a clearer picture of how these technical elements contribute to the specific injury patterns seen in both amateur and elite practitioners [14,16].

A nuanced analysis of the data reveals a divergent injury pattern based on expertise level. We observed a statistically significant difference between professional and amateur dancers, with professionals demonstrating a lower overall risk of injury (*p*-value = 0.037). This protective effect in the professional cohort may be attributed to superior neuromuscular conditioning, refined technique, and better proprioceptive control during complex maneuvers. However, the distribution of injuries varies qualitatively: while amateurs are significantly more prone to shoulder injuries, likely due to technical errors during the learning curve of powermoves, professionals report a higher frequency of wrist injuries (61.8%). This shift suggests a transition from acute, technique-related trauma in novices to chronic, cumulative overuse syndromes in experts. The professional breaker’s wrist is subjected to years of repetitive axial loading in dorsiflexion, a mechanism known to cause dorsal impingement and triangular fibrocartilage complex (TFCC) pathology, identifying a specific area for preventive intervention in elite dancers [13,16].

The analysis of preventive measures yielded results that warrant critical interpretation. Despite 96.9% of participants reporting the execution of warm-up routines, this practice did not show a statistically significant correlation with injury reduction (*p*-value = 0.168). This lack of statistical significance is likely driven by a “prevention paradox” rooted in reverse causality. While the quality and specificity of current warm-up protocols may be insufficient to meet the explosive demands of breaking, it is more probable that the cross-sectional nature of our data captures a reactive behavior: dancers may implement more rigorous warm-ups or stretching only after the onset of chronic pain or a previous injury [5]. Moreover, our finding that 70.10% of dancers train without a coach is clinically significant when viewed through the lens of recent evidence. Brborović et al. explicitly identified the lack of training supervision as a critical risk factor in their analysis of severe injuries [14]. This suggests that the high injury prevalence observed in our cohort may not be solely due to biomechanical load, but also to the absence of technical correction and load management that a qualified professional would provide.

Standard static stretching, often favored by dancers, may not adequately prepare the capsuloligamentous structures for the explosive, ballistic demands of breaking. Similarly, the use of protective equipment (knee pads, helmets, elbow pads) did not correlate with a reduced injury rate (*p*-value = 0.164). While this might imply a lack of efficacy, it is crucial to consider the potential for reverse causality bias, as noted in our results. It is highly probable that dancers begin to utilize protective gear only after sustaining an injury, as a secondary prevention measure or to manage existing pain, rather than as a primary prophylactic strategy. This temporal inversion obscures the true potential benefit of protective equipment, highlighting the need for prospective longitudinal studies to clarify this relationship [4,10].

From a clinical management perspective, the study highlights a positive trend in the Italian context: 78.35% of injured dancers sought professional medical consultation, indicating a high level of health literacy and trust in the healthcare system. This proactive approach aligns with recent findings, which emphasize that timely specialized assessment is critical to optimize conservative management and potentially reduce the need for surgical intervention in sports-related injuries [20]. This contrasts favorably with previous French studies where most dancers did not seek medical attention [15]. The fact that only a small minority reported unresolved issues suggests that when appropriate orthopedic and rehabilitative care is accessed, the prognosis for returning to sport is generally favorable. 

Studying this Italian cohort is crucial for identifying the medical vulnerabilities of a population caught in the middle of a major cultural shift. As breaking evolves from a grassroots, autodidactic activity into an Olympic-level discipline, the development of targeted musculoskeletal care has unfortunately lagged behind. In the Italian healthcare context, where sports medicine is highly structured for traditional disciplines, there remains a critical awareness gap regarding the unique “functional inversion” and high-velocity torque demands inherent to breaking. By identifying the specific injury patterns and the high prevalence of unsupervised training (70.10%) in this population, our research provides the necessary evidence base to develop targeted national clinical guidelines and preventative protocols for orthopedic and physical therapy professionals.

Finally, the role of environmental factors, specifically training surfaces, remains a critical yet complex variable. Our data show extreme heterogeneity in training conditions, with dancers alternating between parquet, concrete, linoleum, and marble. While statistical correlation was not feasible due to this variability, the impact of non-shock-absorbing surfaces (like concrete and asphalt) on the kinetic chain cannot be ignored. The lack of standardized flooring represents a significant extrinsic risk factor that likely exacerbates the traumatic potential of the discipline, contributing to the high prevalence of foot and ankle pathologies observed. 

## 5. Limitation

While this investigation provides a necessary epidemiological baseline for breaking in Italy, its findings must be weighed against several methodological constraints. First, the study’s cross-sectional design relies heavily on retrospective self-reporting, which naturally introduces recall bias. Participants may have overlooked distant or minor injuries, and the absence of objective clinical records to verify these reports makes the data highly dependent on individual recall accuracy. As such, these results should be interpreted with caution.

A significant constraint lies in the sample characteristics. While a cohort of 97 dancers is substantial for such a specific niche, this sample size limits our ability to perform more granular sub-group analyses. Moreover, the study population is quite heterogeneous, spanning ages from 12 to 46 and involving training on surfaces as different as professional parquet and raw concrete or asphalt. This environmental variety acts as a confounding factor; such “noise” in the data is difficult to standardize in a survey-based study and may influence injury mechanics in ways that aggregated data cannot fully capture.

As noted in our results, approximately 37% of respondents were unable to specify the exact duration of their training suspension following a trauma. This lack of precise recall prevents a complete assessment of injury severity—specifically the “time-loss” metric common in sports medicine—making it difficult to definitively distinguish between minor musculoskeletal complaints and more debilitating pathologies.

The distinction between “professional” and “amateur” athletes also requires a careful reading. In the urban dance world, this boundary is often more fluid than in institutionalized sports. Although we categorized dancers based on training volume and experience, this does not always correlate with economic status or the high-level medical support typical of “pro” athletes. Consequently, our professional category likely includes elite performers who still lack the specialized recovery resources of traditional athletes, potentially blurring the risk profile differences between the groups.

Furthermore, the analysis of overuse injuries should consider external cumulative loads. Although we excluded those practicing classical dance to limit cross-discipline interference, we did not control for the participants’ occupational backgrounds. The high frequency of wrist and shoulder issues observed might partially stem from “stacking” breaking mechanics on top of daily occupational strains, whether from heavy manual labor or repetitive office work, that the questionnaire did not account for.

Finally, the recruitment strategy, conducted primarily through social media and international competitions, introduces a clear selection bias. By targeting active competitive environments, we likely captured those who have remained in the sport despite their injuries. This approach may inadvertently exclude individuals who were forced to leave the breaking scene due to chronic pain or career-ending pathologies. Future longitudinal research will be vital to tracking these athletes over time and isolating the specific impact of breaking on joint health.

## 6. Conclusions

This study represents the first epidemiological baseline for breakdancing injuries in the Italian population, confirming the discipline as a high-demand sport with a significant traumatic burden. The findings underscore that the unique biomechanics of breaking, specifically the functional inversion of the kinetic chain during acrobatic maneuvers, creates a distinct traumatic profile that differs significantly from traditional upright dance forms. It appears that current preventive strategies and the largely autodidactic training culture are insufficient to mitigate the risks of both acute trauma and cumulative overuse syndromes. Consequently, clinical management of the breakdancer demands a paradigm shift beyond standard sports medicine protocols. Protecting athlete health in this evolving Olympic sport requires the integration of professionalized coaching with specialized conditioning programs. Based on our findings, these protocols should prioritize scapulothoracic and glenohumeral stabilization to manage the extreme axial loads and high-velocity torque on the shoulder, alongside targeted strengthening of the forearm musculature to enhance wrist resilience against repetitive weight-bearing stress. Future scientific efforts must now move toward longitudinal monitoring to validate sport-specific injury prevention guidelines.

## Figures and Tables

**Table 1:** Demographic differences between professional and amateur dancers.

	Professional Dancers (68 Dancers)		Amateur Dancers (29 Dancers)		*p*-Value
	Media	DS	Media	DS	
Age (y)	26.17	7.88	25.89	8.5	0.85
Height (cm)	170.94	8.19	171.69	9.05	0.63
Weight (Kg)	65.77	9.02	67.34	11.47	0.31
BMI (m/Kg^2^)			22.60		

**Table 2:** Frequency of injuries according to anatomical location in professional dancers and amateur dancers: the following table describes the cases and injury percentages according to the anatomical district, differentiating professional and amateur athletes.

Anatomic District	Total Cases (*n* = 97)		Professional Dancers (*n* = 68)		Amateur Dancers (*n* = 29)	
	Total	%	Total	%	Total	%
Head	13	13.4	10	14.7	3	10.3
Neck	47	48.5	34	50.0	13	44.8
Spine	19	19.6	16	23.5	3	10.3
Shoulder	59	60.8	41	60.3	18	62.1
Elbow	36	37.1	29	42.6	7	24.1
Wrist	56	57.7	42	61.8	14	48.3
Hand	36	37.1	25	36.8	11	37.9
Hip/Thigh	26	26.8	20	29.4	6	20.7
Knee	62	63.9	43	63.2	19	65.5
Ankle	33	34.0	26	38.2	7	24.1
Foot	18	18.6	13	19.1	5	17.2

**Table 3:** Main skills: This table shows what the main skills are trained and how many amateur and professional athletes say they train individual skills.

Skills	Total	Professionals	Amateur
Toprock	38	27	11
Footwork	73	54	19
Freeze	47	35	12
Powermove	59	38	21

**Table 4:** Distribution of injuries by anatomical district and gender: summarizes the frequency of traumatic events classified by specific anatomical location, highlighting the numerical distribution of cases between the male and female populations.

District	Man	Women
Head	13	0
Neck	42	5
Spine	16	3
Shoulder	53	5
Elbow	33	3
Wrist	51	5
Hand	32	4
Hip/Thigh	25	1
Knee	52	10
Ankle	15	3
Foot	8	0

## Data Availability

Data is contained within the article.
